# Experimental evidence of the effect of financial incentives and detection on dishonesty

**DOI:** 10.1038/s41598-022-06072-3

**Published:** 2022-02-17

**Authors:** Mehak Kaushik, Varsha Singh, Sujoy Chakravarty

**Affiliations:** 1 Department of Resource Economics, University of Massachusetts Amherst, Amherst, MA USA; 2 Department of Humanities and Social Sciences, Indian Institute of Technology Delhi, New Delhi, India; 3 Centre for Economic Studies and Planning, Jawaharlal Nehru University, New Delhi, India

**Keywords:** Psychology, Human behaviour

## Abstract

We revisit two fundamental motivations of dishonesty: financial incentives and probability of detection. We use an ability-based real effort task in which participants who are college students in India can cheat by over reporting the number of puzzles they could solve in a given period of time. The puzzles are all unsolvable and this fact is unknown to participants. This design feature allows us to obtain the distribution of cheating outcomes at the individual level. Controlling for participant attributes, we find that introducing piece-rate financial incentives lowers both the likelihood and magnitude of cheating only for individuals with a positive probability of detection. On the other hand, a decrease in the probability of detection to zero increases magnitude of cheating only for individuals receiving piece-rate incentives. Moreover, we observe that participants cheat significantly even in the absence of piece-rate incentives indicating that affective benefits may determine cheating. Finally, an increase in own perceived wealth status vis-à-vis one’s peers is associated with a higher likelihood of cheating while feeling more satisfied with one’s current economic state is associated with a lower magnitude of cheating.

## Introduction

For almost all of us, material advancement is contingent on a measure of individual performance. Examples of performance yardsticks include meeting company targets, acquiring requisite expertise and job experience, publishing academic papers, attaining high scores in admission tests, athletic achievements, etc. In this context, deception in the form of deliberate lying or using unfair means to misrepresent one’s ability, performance or intelligence to obtain material and status-based rewards is widespread^1^. For example, in the Indian education system, Sheriff et al.^2^, Sivagnanam et al.^3^, Babu et al.^4^ and Gitanjali^5^ find significant numbers of medical students claiming that they cheated in their university examinations to get higher scores. Moving from education to job markets, a recent survey by TribePad finds that among candidates who use application tracking systems for jobs in the UK, over 85 percent “optimize” their resumes by doctoring their listed skills and lying about experience^6^. Using data on Indian job seekers to gauge the level of applicant fraud in India from 2012–2016, a First Advantage survey report finds that for every 100 candidates screened, 10–12 have one or more discrepancies in their stated qualifications (First Advantage^7^. Thus, it seems clear that while performance or evaluated ability is rewarded by principals, the means of achieving these productivity targets are often imperfectly monitored for agents providing a scope for dishonest acts on the part of the latter. But do all individuals cheat maximally when they get an opportunity? In this paper, we revisit two fundamental motivations of cheating i.e., incentives and probability of detection. Our experimental design uses an ability-based real effort task and allows us to obtain individual and joint effects of these factors on the individual level of dishonesty.

Incentives and detection of corrupt and dishonest practices of individuals are central to the economic approach to dishonesty^8,9^ and their study is crucial to the understanding of principal-agent relationships. These in turn have strong implications particularly for the detection of corrupt practices related to the governance of organizations^10^. However, several later studies in the individual decision-making paradigm such as Mazar et al.^11^, Fischbacher and Follmi-Heusi^12^, Balasubramanian^13^, Kajackite and Gneezy^14^ and Hilbig and Thielmann^15^ study dishonesty in the laboratory by varying incentives but do not find consistent incentive effects. Furthermore, Mazar et al.^11^ experimentally vary the probability of detection of cheating and find that it does not significantly affect dishonesty. Overall, the literature is characterized by non-convergent effects of both probability of detection and financial incentives on dishonesty. A plausible reason for these non-convergent results may be that no other study except for Kajackite and Gneezy^14^ has attempted to systematically cross incentives with detection probability, using the same laboratory tasks. Different studies check one or the other motivation using different task contexts (puzzle tasks, die rolls, coin flips), reward magnitudes and experimental protocols (online, laboratory, field) making results difficult to compare. A second serious consequence of not crossing these two motivations is that it forces us to theoretically imagine the effect of one motivation to essentially be uniform over values of the other. Thus even in the context of a single task, non-crossed studies do not allow us to observe effects arising out of interactions of different levels of the two variables. Studying these interactions would thus aid in refining and extending existing theories of dishonest behaviour. Our primary contribution consists of using one set of laboratory tasks (which fixes the context) and systematically testing the level of dishonesty in the presence or absence of financial incentives and detection in a two-by-two design.

As a secondary contribution, our experimental design allows us to obtain magnitude of dishonesty at the individual level. In the existing literature, dishonesty is measured almost always by comparing the average real-effort task performance of a cohort that is allowed to cheat with one that is not^11,16^ or in comparison to a uniform distribution: e.g.—die-roll^12,14,17^ or coin-flips^13^. Studies using uniform distributions or cohort level statistics provide an estimate of the magnitude of cheating but are not informative in estimating the proportion of cheaters in a cohort. In contrast, our design allows us to obtain a direct measure of dishonesty at the individual level. Further, as our data is at the individual level, it allows us to control for individual specific attributes when estimating the impact of incentives and detection probability on cheating and also correlate individual specific attributes with levels of cheating.

Participants in our experiment, who are college students in New Delhi, India are given puzzle tasks that test spatial logic skills to attempt within a stipulated time limit. They then either leave their problem sheet at the experiment venue or shred it before self-reporting the number they purportedly solved. Depending on the treatment condition they are in, they receive a flat show-up fee and a piece-rate payment for every puzzle they claim they solved, or simply a flat show-up fee. What participants do not know is that none of the puzzles actually have solutions. Thus the self-report gives us an accurate measure at the individual level of their level of dishonesty in over-reporting the number of problems they solved. We are then able to associate individual observation on cheating with treatment conditions (rewards, probability of detection) as well as a number of individual socio-demographic attributes.

### Survey of literature: cohort versus individual level studies

According to Gary Becker’s Simple Model of Rational Crime (SMORC^8,9^), a rational individual would commit a crime as long as the marginal benefit exceeds the marginal cost of committing one, and cheat to the maximum extent possible. However, in a pioneering laboratory study of dishonesty in individual decision making using real-effort “matrix” addition puzzle tasks, Mazar et al.^11^ obtain that independently increasing incentives and probability of detection have no significant influence on cheating. On the other hand, priming that emphasizes attention to standards such as recalling the ten-commandments and signing an honour code or allowing for a more malleable moral categorization of cheating, decrease and increase cheating respectively. Their results foreground a moral balance theory^11,18^ in contrast to the purely economic model of cheating, where in addition to external rewards and detection, the decision to be dishonest is also influenced by an internal moral standard. As a result, individuals may be self-serving in order to gain rewards but only up to an internally defined threshold. Yaniv and Siniver^16^ extend^8,9^ to incorporate social motives such as shame and find that these could provide internal curbs that limit maximal cheating.

This “incomplete cheating” paradigm is seen in studies at the cohort level. Several of these compare behaviour at the cohort level against a uniform distribution. A common method is to have participants report the realization of a private die-roll or rolls. The dice used have different numbers of sides and are sometimes implemented on computer interfaces. Studies that use die rolls include Shalvi et al.^19^, Lewis et al.^20^ Fischbacher and Follmi-Heusi^12^, Kajackite and Gneezy^14^, Hao and Houser^21^, Hilbig and Thielmann^15^ and Charness et al.^17^. A second method consists of participants privately flipping a coin or coins either physically or on a computer and self-reporting the outcome and has been implemented by Abeler et al.^22^, Pascual-Ezema et al.^23^, Hugh-Jones^24^ and Balasubramanian et al.^13^ and Pate^25^. Additionally, there are studies that ask participants to self-report the number of addition problems (e.g.-matrix task) they solved. These studies typically compare average self-reports of cohorts that are asked to shred or recycle their sheets to those from comparable cohorts where answers are checked^11,16^. In a meta-analysis of 393 experiments that use these tasks, Gerlach et al.^1^ find that the average estimated dishonest behaviour is greater for die-roll and coin flip tasks than for matrix tasks, but only a few individuals cheat to the maximum extent possible in both. This incomplete cheating is also noted in Abeler et al.^26^ in their meta-analysis of 90 studies that use the self-reporting of a die-roll.

The literature is dominated by a large number of studies that attempt to measure dishonesty at the cohort level as individuals may be careful to not cheat if they knew they were being observed. Those with large samples that compare cohort averages against a theoretically known uniform distribution are often adequate to capture treatment effects in a simple non-invasive way that does not significantly bias the decision maker’s preferences. However for smaller samples, realizations may not follow theoretical expectations leading to noisy assessments regarding dishonesty. Moreover, cohort studies that compare two groups using a between subjects design (e.g.—making one group shred and checking the performance of another) may potentially run into an issue regarding the comparability of groups. A smaller number of studies capture cheating at an individual level and make it possible to independently study both the extensive (number or proportion of cheaters) and intensive (the magnitude of cheating among cheaters) margins of cheating. Thus these studies allow us to identify in a sample whether many individuals cheat by a small amount or whether a few “bad apples” cheat maximally to boost the group average. In a field experiment in Be’er Sheva Israel, Maharabani^27^ who studies whether or not individual shopkeepers and taxi drivers over charge a blind customer more than a sighted one, finds no evidence of opportunistic maximal cheating on the part of these individuals. Friesen and Gangadharan^32^ use the same real-effort matrix task as Mazar et al.^11^ and in their only experimental condition matrix sheets are collected from participants and checked later. They find that among participants who are university students in Australia, about one third cheat and only a quarter of the dishonest take the maximal amount possible. Van Der Zee et al.^28^ who identify cheating on an individual level, conduct experiments where participants recruited through M-Turk are asked to answer multiple choice grammar and semantics questions where they have an opportunity to cheat. They also find that individuals do not cheat by the maximum amount possible. Tzini and Jain^29^ use observation on self-reported performance on a quiz adapted from online IQ tests and find that approximately 85 percent of individuals cheat but the average cheating is well below maximal cheating. Thus, cheating appears incomplete or non-maximal according to individual level experiments in the literature.

Though there are numerous studies that document incomplete cheating using specific reward and detection levels, there are very few experimental studies that systematically vary incentives and/or the probability of detection to check their effects on cheating in the laboratory.

### Varying incentives and probability of detection

Keeping detection probability at zero (subjects shred answer sheet), Mazar et al.^11^ et al. compare cheating in the matrix task at piece rates of $0.50 and $2.00 and find that this decreases cheating but not significantly. They also report that they conduct sessions that vary piece rate incentives between $0.1, $0.5, $2.5 and $5.0 get a similar result. Using a six sided die roll task, Fischbacher and Follmi-Heusi^12^ multiply realization specific payments (CHF 0–5) by a factor of three and find that it does not alter reported die-rolls significantly. In an online study using Amazon M-Turk participants from India, Balasubramanian et al.^13^ employ 6 flips of a fair coin and pay a piece rate reward that increases from $ 0.50 to $ 5.00 in 0.50 increments. They find that over reporting increases when the piece-rate goes up from $ 0.50 to $ 3.00 and decreases at higher piece-rates. Hilbig and Theilmann^15^ conduct a multi-period adaptive die-roll experiment and find that overall about 60 percent of participants fail to reach the maximum incentive as incentives increase. Additionally, they identify two subsets of participants who respond to financial incentives: ‘‘corruptible individuals” (cheating increases with rewards) and ‘‘small sinners” (cheating decreases with potential rewards). Van Der Zee et al.^28^ use grammar and semantics tasks in an online experiment and find that the level of cheating is invariant to the presence or absence of financial gains. Finally, in keeping with rational economic motivations for cheating, the absence of incremental financial incentives from cheating is associated with negligible cheating in the die-roll experiments of Charness et al.^17^. However, from studies by Ruedy et al.^30^, Van der Zee et al.^28^ and Singh and Chakravarty^31^ it appears that other non-pecuniary psychological motivations may provide a utility boost from cheating to the decision maker in the absence of financial incentives. Besides Mazar et al.^11^, studies mentioned earlier do not vary the probability of detection. The latter implement a quiz with 50 general knowledge questions and a fixed piece rate of $ 0.10 but vary the probability of detection by implementing three cheating conditions. In the “no recycle” condition, participants transfer their answers from their test sheet to a bubble sheet already pre-marked with the right answers. They have to then return both test and bubble sheets to the experimenter and claim payment on the basis of the bubble sheet. The “recycle” condition is identical to “no recycle” except that participants shred the test sheet and hand over only the bubble sheet for payment. Finally, “recycle + ” is identical to recycle except that participants shred both test and bubble sheets and pay themselves from a jar. Thus, probability of detection declines over the three conditions. Interestingly, they find that this does not significantly alter cheating.

Our study is closest to Kajackite and Gneezy^14^, which is the only study apart from ours that attempts to vary both incentives as well as probability of detection. However, they do not use an ability based task like we do and obtain only cohort level estimates of cheating. They employ a six-die roll task that pays $X (X = 1, 5, 20, 50) if a realization of 5 is reported. In the standard self-report of the die-roll task, increasing incentives does not significantly change the level of dishonesty. Additionally, and differently from Mazar et al.^11^ and our study, they do not objectively alter the detection probability. Instead, they attempt to vary the perceived detection probability by implementing a framing of the participant self-report they call the “mind game.” Accordingly, instead of reporting the realization of a die roll as is standard in the experimental literature, participants are asked to think of a realization (and not report it), and verify whether or not the same number came up in the die roll. They find that at all reward levels enacting the mind-game increases dishonest reporting. However, even in the mind game they do not obtain a consistent effect of increasing incentives on cheating, which increases non-monotonically when the reward level increases from $1 to $50. Specifically, dishonesty increases at the $5 level, falls at the $20 level and then rises again at the $50 level. The difference in cheating between reward levels of $5 and $ 50 is the only change found to be statistically significant.

### Effect of gender, age and ability on dishonesty

As in our study, there are other studies from psychology and economics which correlate individual attributes with dishonesty in different task contexts. Friesen and Gangadharan^32^ who use the matrix task and Cason et al.^33^ who study laboratory audit tournaments find that men have a higher likelihood of being dishonest. On the other hand, Yaniv et al.^34^ who use a trivia quiz and Pate^25^ employing a coin flip task finds no difference in cheating between genders. Among meta-studies, Whitley et al.^35^ conduct one on the effect of gender on academic cheating and find that the gender difference in behavior is small and sensitive to cultural contexts. On the other hand, a meta- study conducted by Gerlach et al.^1^ finds that men cheat more and younger participants cheat more than older participants. The latter is also verified in the tax compliance experiments of Alm et al.^36^. The effect of ability on cheating is similarly ambiguous: Miller et al.^37^ find that academic cheating is higher for lower ability children while Ruffle and Tobol^38^ confirm this in their die-roll experiments on Israeli soldiers. On the other hand, Cason et al.^33^ find the opposite, i.e.—higher ability individuals cheat more. This is verified in the context of actual examinations from an undergraduate psychology course by Ottaway et al.^39^, an in-class repeated trivia quiz by Yaniv et al.^34^ and a creative performance task by Alan et al.^40^.

### Effect of wealth and aspirations

There is a small literature on the effects of social status and wealth aspirations on dishonest or unethical behaviour. Both subjective and objective social status predict proclivities towards unethical acts according to seven studies using both laboratory and naturalistic methods by Piff et al.^41^. Individuals who are or feel themselves to be of a higher economic status are more likely to cut off vehicles at stop signs, take candy meant for children and over report the realization of a die roll to make more money. Higher cheating has also been documented in elementary school children of higher socio-economic class by Alan et al.^40^. On the other hand, Andreoni et al.^42^ who study a natural field experiment in the Netherlands, find that there is no difference in ethical behaviour among the rich and poor. There is no theory that explicitly links economic aspirations, measured by the difference between current and desired wealth states, to dishonest actions though Merton^43^ and Lerman^44^ do allude to aspirations sometimes driving dishonest behaviour, especially those stemming from social comparison. There are no laboratory studies known to us that examine the effect of economic dissatisfaction on the proclivity to cheat.

In addition to individual decision-making environments that we focus on in this study, dishonesty is examined in multi agent game theoretic interactions where cheating imposes payoff externalities on other players. Here factors such as reputational concerns, other regarding preferences and conditional cooperation may influence the level of cheating^26^. For more comprehensive documentation of dishonesty in both individual choice and game theory experiments see Rosenbaum et al.^45^, Jacobsen et al.^46^ and Gerlach et al.^1^, which review numerous dishonesty studies from both psychology and economics and try to offer a unified perspective on cheating behaviour.

## Method

### Experimental design

We conduct our experiment with 284 undergraduate students from ten institutions of higher education located in middle-income neighbourhoods of south and southwest Delhi and the western suburb of Gurugram within the national capital region of Delhi. These comprise seven undergraduate colleges affiliated to a large public university, two public universities and one private university. The institutions all have a diversified student pool in terms of socioeconomic class but most students would be classified as middle or upper middle-income. Experimenters first contact an administrator or principal in each college or university. If permission to recruit is given, research assistants visit college classrooms and verbally announce an opportunity for students to make some money by participating in a project on decision-making. No specific details are provided at this stage. Interested participants contact the research team by email and the first 30 of these individuals in each institution are invited to participate. The participating subjects in our sample are from Business, Mathematics and several disciplines within the Humanities and Social Sciences. The average age of participants is 19.5 years and 55 percent are women (see Table 3 for some descriptive statistics of our sample and Supplementary Table S2 for a break up of these descriptive statistics by treatment group). Each session consists of a questionnaire, a puzzle solving task and the self-reporting of performance. The sub-sections below provide details of treatments, experimental tasks and session protocol followed in our experiment.

### Treatment conditions

Our experiment, which has participants self-reporting the number of puzzles they solved, uses a between-subjects design and examines preference for dishonesty in a two by two (2 × 2) design of our treatment variables: reward and probability of detection. The reward variable has two levels namely, Piece Rate payment [P] and No-Piece Rate payment [NP]. The probability of detection variable consists of two levels: Shred [S, zero probability], where participants destroy evidence of cheating, and No- Shred [NS, positive probability] where they do not. We run experimental sessions with all possible combinations of treatment levels, which are NP/S, NP/NS, P/S and P/NS. Due to this, we are also able to test for the joint effect of reward and detection on dishonesty. Descriptions of these four treatments and the number of participants for each treatment are given in Table 1.

**Table 1:** Number of observations for four treatment groups.

Treatment groups	No. of participants	Show up (flat) fee	Piece Rate payment	Shred puzzle sheet
No Piece-rate/No Shred (NP/NS)	60	Rs. 250	None	No
No Piece-rate/Shred (NP/S)	82	Rs. 250	None	Yes
Piece-rate/No Shred (P/NS)	60	Rs. 250	Rs. 50 per reported solved	No
Piece Rate/Shred (P/S)	82	Rs. 250	Rs. 50 per reported solved	Yes

Participants in all sessions (irrespective of treatment) receive a flat show up fee of Rs. 250. In the P conditions (P/S and P/NS), they are additionally paid piece-rate of Rs. 50 per puzzle they report to have solved. Thus, payoff for a participant in the P condition is given by 250 + 50*X, where X is the number of puzzles they purportedly solved. Accordingly, a participant can report that he or she solved 10 problems and receive the maximal payoff of Rs. 750. The latter is approximately equivalent to US$ 42 using PPP exchange rates between India and the USA in 2017 (1 US$ = Rs. 17.81), as given in the World Bank, International Comparison Program Database (see Supplementary Table S1 for URL). These stakes are high given the ages of participants, their earning potential and the duration of the session. Our experiment does not carry any explicit punishments or sanctions for cheating as these would be prohibitively difficult to enact post session and may violate anonymity of participants. Moreover, from the results of Nagin and Pogarsky^47^ and several studies in the criminal deterrence literature they survey, dishonest behaviour is more sensitive to changes in detection probability and varies negligibly with severity of sanctions.

For each college, the 30 participants are randomly allocated between the P and NP conditions, in two sessions of 15 participants each. All participants from four colleges are randomly allocated to the NS treatment while all participants from the six remaining colleges are allocated to the S treatment. This gives us a total of 120 participants who turn in their puzzle sheets after they self-report their score. The cohort size is 30 for 9 institutions. In the tenth, which is a college allocated to the S condition, due to subjects not showing up we run the experiments with 14 participants equally divided over the two conditions. Except for this one institution, there is no attrition and all other participants who were invited for a session participated in it.

### Session protocol

Our experiments are carried out in accordance with relevant guidelines and regulations prescribed by the Institutional Ethical Review Board (IERB) of Jawaharlal Nehru University, who approved the experimental protocols we employ in our study. Informed consent is obtained from all participants**.** For the few participants who are below 18, we do not independently collect any family contact information as this would violate anonymity in our experiment. The college administrations had this information and obtained all necessary clearances before giving us the go ahead to run paid sessions with recruited participants on college premises. Sessions are conducted in college classrooms. Participants are seated spaced out over the room and are not allowed to communicate with one another during the experiment. We begin by providing each participant with a unique and anonymous identity (ID) number. At this stage, participants are informed that they would receive a flat show up fee of Rs. 250. Additionally, only in Piece Rate payment conditions (P/S and P/NS), participants are informed of the possibility of earning more in the experiment. As a next step, participants are given a questionnaire to fill in with attitudinal and demographic information. The questionnaire is a part of a larger project on dishonesty and aspirations and is not reproduced in full here but can be obtained from the authors on request. The unique IDs are later used to correlate responses in the questionnaire to those in the experiment. After the questionnaire is completed and collected, participants are informed about the subsequent puzzle-solving task. It is at this stage that participants in the Piece Rate payment conditions (P/S and P/NS) are given detailed information about additional earnings.

We minimize the effect of speculation or suspicion regarding difficulty/solvability of the problems from contaminating our participant pool by removing puzzle sheets and all instructions from participants before they leave the experiment venue. Furthermore, we do not allow participants to bring in cell phones or devices of any kind, which may allow them to take a photo of experimental instructions and puzzle sheets to study later or communicate to other potential participants. The P and NP sessions at each institution are conducted simultaneously in spatially separated classrooms. Moreover, we conduct only one set of parallel sessions at an institution to ensure that participants in earlier sessions do not inform later arriving individuals regarding the difficulty level of the tasks.

### Experimental tasks

In the puzzle solving task, all participants are given a set of ten (10) puzzles to be attempted in five (5) minutes. These ten problems (including several mazes) test spatial ability and do not test mathematical or linguistic skills. See Supplementary Information for the puzzle sheets, instructions and self-report card used in our study. Supplementary Table S1 lists sources for these puzzles. An important reason for using ability based tasks rather than more routine addition and die-roll tasks is that dishonesty over these have been much less explored in the experimental literature. On the other hand, self-serving misrepresentation of ability and intelligence is commonly observed in the academic and professional world as we highlight in our introduction. Thus we feel that it is important to experimentally examine preferences for misreporting productivity in tasks that signal ability. Second, using these tasks also allows us to identify affective benefits^30^ that may potentially drive dishonest behaviour as we have treatments in which there are no piece-rate financial rewards.

**Figure 1:**
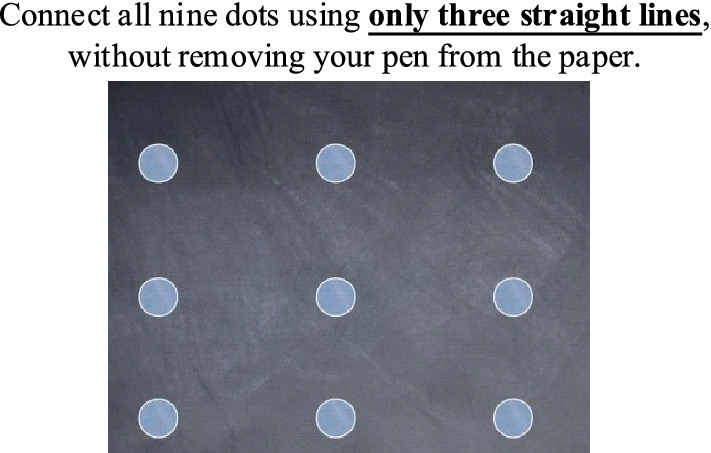
Example-Q4. *source* Mikevanhoozer.com. Modified by authors using MS Paint/Word.

**Figure 2:**
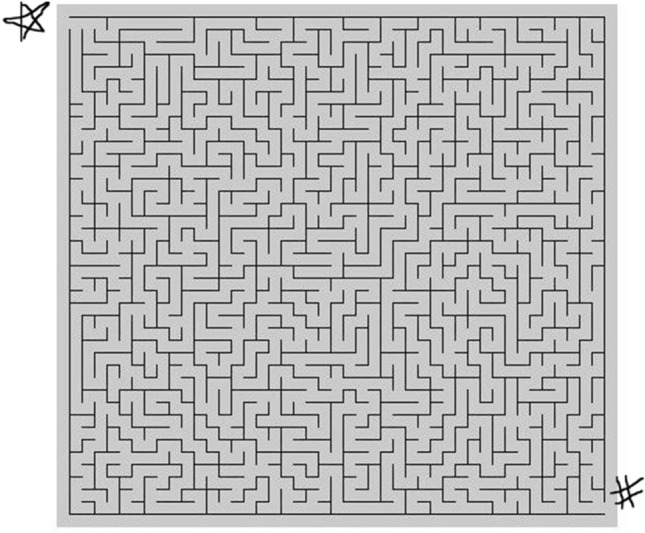
Example- Q7. *source* https://mazegenerator.com. Modified by authors using MS Paint/Word.

Two of the ten puzzles (Q4 and Q7) have been reproduced above in Figs. 1 and 2. The type of puzzles used in our experiment offer two important features necessary for our experiment. First, it is easy for participants to self-determine whether they solved a puzzle or not. For example, in the maze type puzzle, one can effortlessly determine whether they successfully reached the other end of the maze or not. This greatly reduces the possibility of incorrectly attributing an unsolved question as a solved one, something that can occur in case of mathematical problems. Second, all the puzzles used in the experiment are unsolvable. However, this information is unknown to participants. Unsolvable puzzles have also been used to develop a measure of dishonesty by Daumiller and Janke^48^. This design feature allows us to accurately measure dishonesty at an individual level, and at the same time to not have to devise a method to observe what the specific responses are for our participants who are asked to shred their puzzle sheets. If the puzzles had solutions, the surveillance that we would have to institute to observe task productivity would significantly complicate our experimental protocol and create potential for participants to unravel our procedures. Here in our quick and simple protocol without surveillance, participants are given just 5 min to attempt 10 puzzles, and it is almost impossible for them to guess their unsolvable nature. Once the time for the puzzle task elapses, participants who are in Shred (NP/S and P/S) conditions are asked to privately shred their puzzle sheets using the shredder provided at the venue. Hence, they are able to fully convince themselves that their sheets cannot be scrutinized by any experimenter at any time. The procedure remains the same for participants in the No Shred (NP/NS and P/NS) conditions except that they are asked to hand over their puzzle sheets to an experimenter instead of destroying them. These puzzle sheets are put away and not scrutinized any time during the session. There is no evidence that even a single participant figured out that the puzzles were unsolvable though if it had been the case for any of them it would have the potential to bias their response. It is difficult to a-priori conjecture whether this bias would work to make them more careful or increase their self-report as some kind of retaliatory gesture as they had figured out the information purposely kept from them by the experimenter.

### Self-Report and payment

All participants irrespective of treatment are then privately asked to self-report their score on self-report cards. Payment to all participants is done privately and in cash just outside the classroom by an assistant of the experimenter, who also collects their self-report cards. Participants are paid a flat payment of Rs. 250 (US $14 at PPP exchange rate) in the No-Piece Rate (NP/S and NP/NS) conditions and are paid by multiplying their self-reported score with Rs. 50 if they belong to the Piece Rate (P/S and P/NS) conditions in addition to the flat payment of Rs. 250. On average a participant in a P session earns Rs. 304 (approximately US$ 17 at PPP exchange rates).

### Omission of information

An important objective of our research is to identify dishonesty at the individual level. Thus, it is important to keep the unsolvable nature of our puzzles undeclared to the participants. Any information regarding solvability of the puzzles would lead participants to not reveal their true preferences for cheating, effectively causing us to lose experimental control. This form of “ill-defined experiment” has been documented in Hey^49^ where subjects are not given all information pertaining to the decision at the point of decision-making. Hey^49^, Hertwig and Ortmann^50^, Krawczyk^51^, Cooper^52^ and Cason and Wu^53^ clearly distinguish this type of economy of information provision from active “deception” where subjects are purposely manipulated by giving them incorrect information. Furthermore, Cason and Wu^53^ and Charness et al.^54^ define the procedural omission of details pertaining to the true purpose of an experiment as a “grey area” as many published studies in experimental economics employ design innovations that include omitting to mention the exact way pairing occurs between periods and including random restarts which were previously unannounced (Wilson^55^). See also Bonetti^56^ and McDaniel and Starmer^57^ for early systematic discussions on the pros and cons associated with not giving full information or giving incorrect information to subjects. The debate regarding the extent to which withholding information or incompletely specifying the laboratory institution contaminates subject pools is however an ongoing one.

Studies that actually collect experimental and survey evidence regarding the behavioural consequences of deceiving participants display considerable heterogeneity: Barrera and Simpson^58^ and Krasnow et al.^59^ find that participants’ suspicion regarding experimental protocol is not clearly related to past experiences of deception, and that there are no consistent behavioural differences between suspicious and credulous participants. Krawczyk^60^ finds that certifying to participants that there is no deception employed in the laboratory causes a lowering of self-reported suspicion but no significant differences in observed behaviour. On the other hand, Jamison et al.^61^ find significantly different likelihood of return to play after being deceived and a difference in the behaviour of the previously deceived in risk aversion tasks. Thus they provide some qualified support for the proscription of deception. Overall Krawczyk^51^ and Charness et al.^54^ find that concern regarding deceiving subjects is stronger for researchers than for subjects. Cooper^52^, p.113) suggests that for an experiment, the simultaneous satisfaction of four circumstances: high value of results, prohibitive difficulty in operationalization without deception, minimal risk to participants and post experimental debriefing of participants, create a situation where the benefits from adopting a particular procedure outweigh the costs. Though we cannot obviously claim that our procedures have no impact whatsoever on contamination of subject pools for future researchers, we note that omission of information in our experiment allows us to collect individual level data on dishonesty preferences which is rare in the literature and would be prohibitively difficult to collect without it. Moreover, our experiment is conducted with great care taken to ensure anonymity of responses and poses no risk arising from reputational consequences for participants. Finally, individual participants are fully debriefed after completion of all sessions as to the true purpose of our experimental study and it is reiterated that their data contain no personal identifiers.

### Minimizing social desirability bias

To minimize perceived experimenter demand effects that would skew decision making towards social desirability^62^, research students from different institutions conduct sessions and no faculty member is involved in the data collection protocol. Before they provide consent, participants are informed that their responses are strictly confidential and would never be disclosed to anyone with any identifying information. We try to create an environment where none of the participants would feel that the decision to cheat would lead to any reputational consequences for their academic careers or personal lives. These reputational factors are deemed to be important in lowering an agent’s preferences towards dishonesty as documented by Hao and Houser^21^ and Yaniv and Siniver^16^.

### Hypotheses

Our experiment captures cheating at the individual level and allows us to study if cheating occurs as small deviations from honesty for a large number of individuals or from small groups of individuals who display high levels of dishonesty. In this context, the original theoretical economic approach to dishonesty argues that individuals will cheat to maximize their monetary earnings when they cannot be detected. However, this is questioned by several experimental studies from economics and psychology. Accordingly, we frame our first null hypothesis as:

Hypothesis 1**:** An Individual who is paid piece rate incentives cheats by the maximal amount when he or she cannot be detected.

From the literature surveyed we find that results on the extent of dishonesty are non-convergent over monetary incentives and probability of detection and that the absence of incremental monetary incentives is associated with negligible cheating. The null hypotheses regarding incentives and detection probability that we test in our study take the following form below:

Hypothesis 2: Individuals do not cheat when incremental (piece-rate) financial incentives from doing so are absent.

Hypothesis 3: The level of dishonesty proxied either by its extensive margin (proportion of cheating individuals), its average magnitude or its intensive margin (average magnitude among cheaters) is invariant to the presence or absence of piece-rate financial incentives.

Hypothesis 4: The level of dishonesty proxied either by its extensive margin (proportion of cheating individuals), its average magnitude or its intensive margin (average magnitude among cheaters) is invariant when detection probability is increased from zero to a positive value.

We explore the association between feeling wealthier than one’s peers and of feeling of satisfaction with one’s current economic situation on the likelihood and magnitude of cheating among our participants. As economic status has been sometimes seen to increase dishonesty and sometimes not, we frame our null hypothesis conservatively as:

Hypothesis 5: Feeling Wealthier than one’s peers is not associated with the level of dishonesty proxied either by its extensive margin, its average magnitude or its intensive margin.

Finally, as there are no experimental studies known to us regarding the link between economic aspirations and dishonest behaviour, we frame our hypothesis conservatively as:

Hypothesis 6: Dissatisfaction with one’s current economic state is not associated with the level of dishonesty proxied either by its extensive margin, its average magnitude or its intensive margin.

## Results

### Aggregate cheating

**Figure 3:**
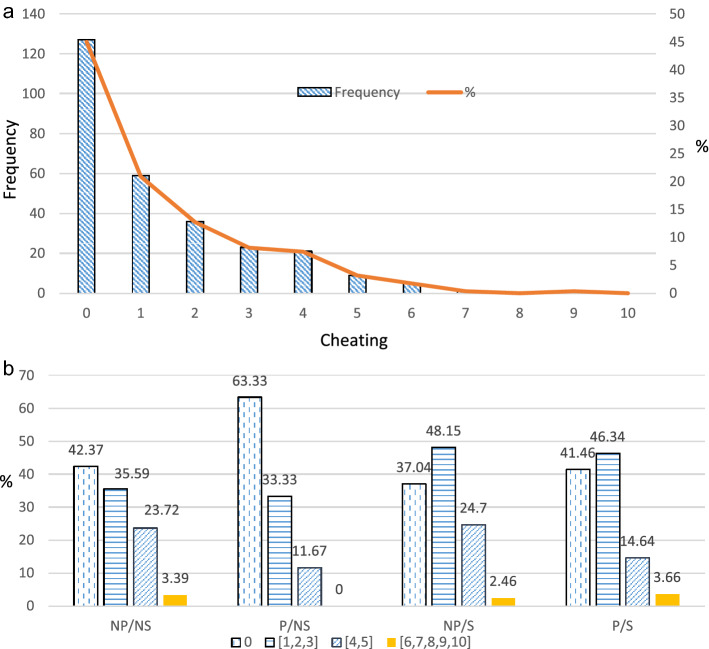
(**a**) Distribution of cheating (aggregating across treatments). (**b**) Distribution of cheating ranges (over different treatments).

Figure 3a presents the distribution of honest and dishonest reporting aggregated over all our treatment conditions. Two participants out of 284 do not self-report the number of problems they purportedly solved, thus their data are unusable. At the extensive margin, approximately 55 percent of the subject pool (155 out of 282 usable responses) lie about the number they solved, i.e. -report solving more than zero problems. Of these 155 dishonest responses, approximately 76 percent of participants report solving 1, 2 or 3 problems but less than 5 percent report solving more than five problems. Further, in our experiment no one cheats by the maximal amount possible which is to report that they solved 10 puzzles.

Figure 3b presents proportions of honest responses (cheating magnitude = 0) and small (1–3), medium (4–5) and large (6–10) ranges of cheating magnitudes differentiated by treatment. Notice that the small category accounts for the largest proportion of cheaters and the large cheaters group represents less than 4 percent of the sample separately for each treatment condition. Aggregating over all treatments, the average likelihood of cheating by a small amount is significantly higher than that of cheating by medium or large amounts (Wilcoxon test, p-values = 0.0000, 0.0000 respectively). Friesen and Gangadharan ^32^ whose experiment is similar to our P/NS treatment find that that close to 10 percent of individuals cheat by the maximum amount possible. In contrast, our results largely conform to that of minor transgressions of mostly honest people as highlighted in Ariely^63^. A design feature of both Friesen and Gangadharan^32^ and Yaniv and Siniver^16^ who also use ability puzzles is to pre-pay participants the maximal amount. Participants then have to return money that they are not owed back to the experimenter. We conjecture that perhaps the endowment effect which is a manifestation of loss aversion^64^ contributes to participants keeping back a larger amount. Grolleau et al.^65^ and Schindler and Pffateicher^66^ find that people cheat more to avoid a loss.

The average reported number of solved puzzles across all treatments is 1.33 problems, which is different from zero at the 1 percent level (Wilcoxon test, two-tailed *p*-value = 0.0000). So, on average over all our treatments, participants report a little over 1 problem more than they could do (truthful reporting is zero in all our treatments), which is in the range of what Mazar et al.^11^ find. At the intensive margin, considering only the cohort over all treatments who cheat (N = 155), we find that the average magnitude of cheating is higher at 2.42.

### Treatment effects on cheating

**Figure 4:**
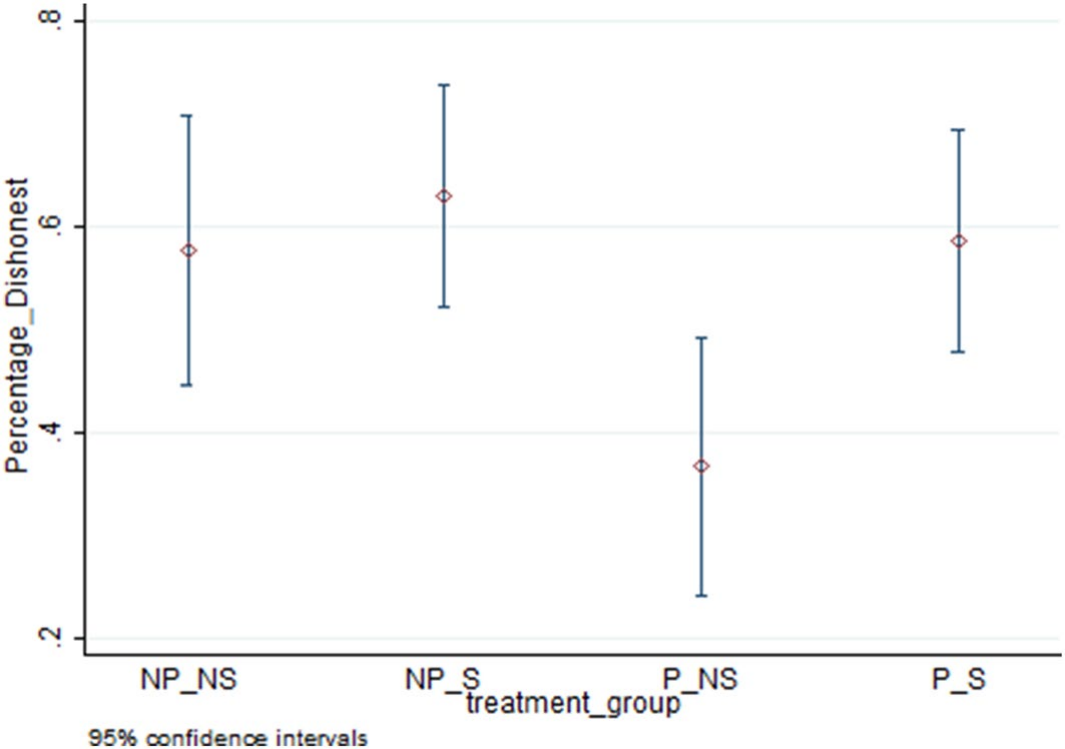
Percentage of individuals who cheated by treatment combination.

**Figure 5:**
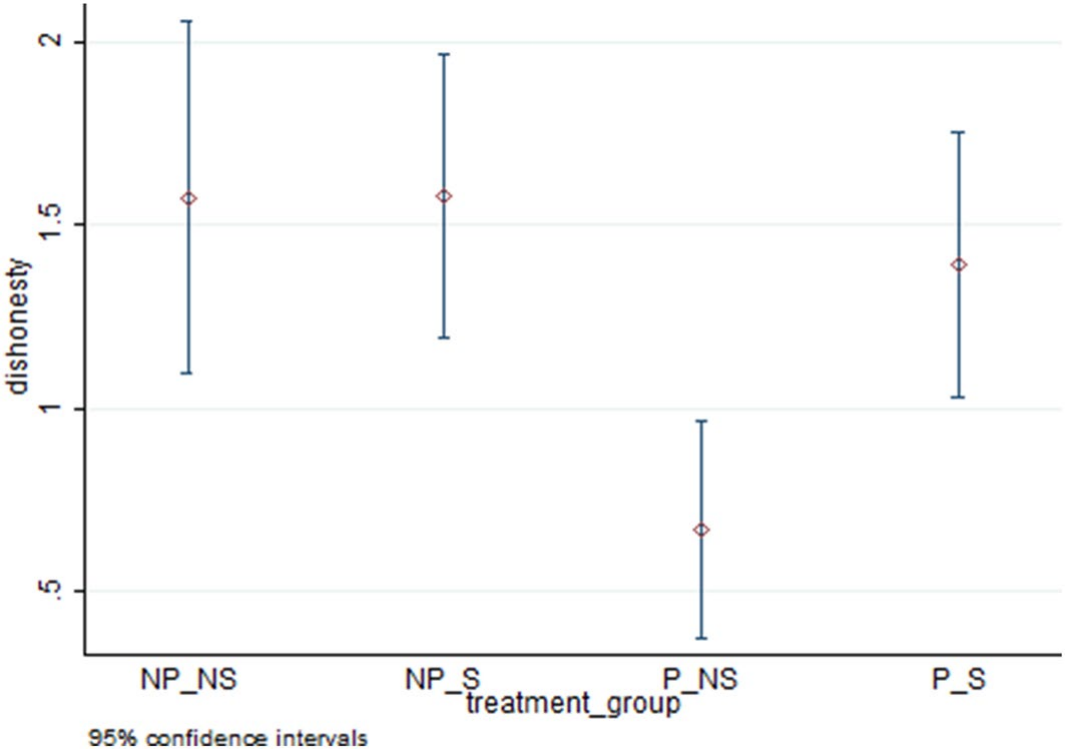
Average reported as solved by treatment combination (all individuals).

**Figure 6:**
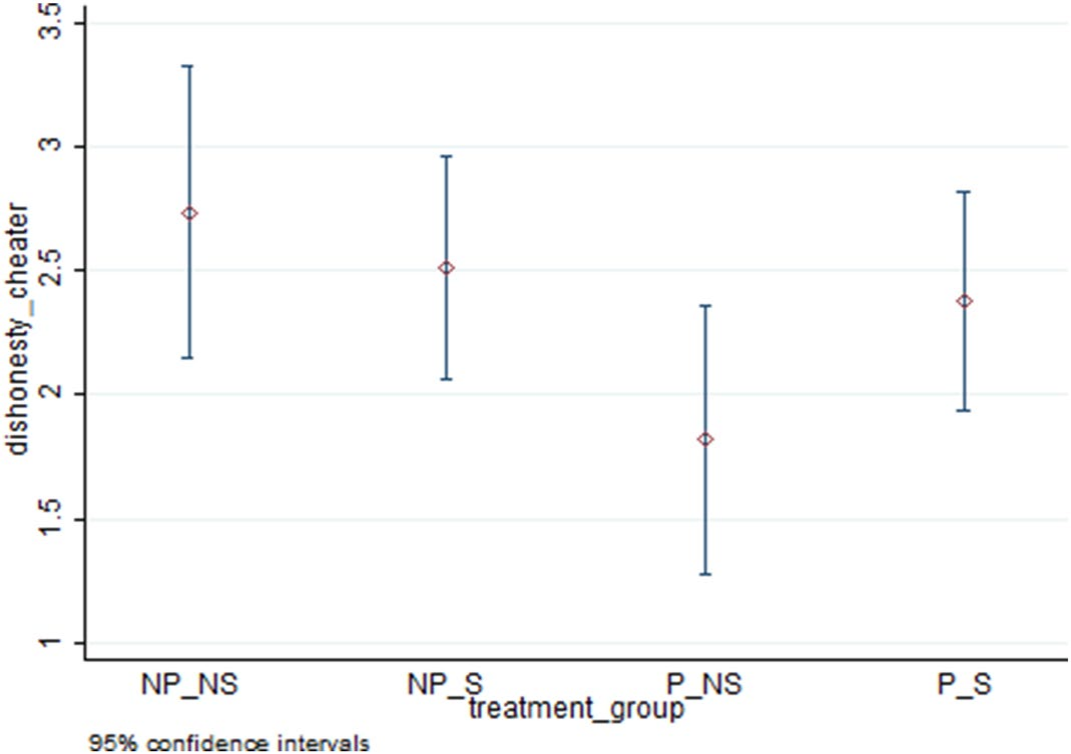
Average magnitude of cheating by treatment combination (only cheaters).

The top four rows of Table 2 present treatment-wise averages for our measures of cheating. These are represented by the proportion who cheat (extensive margin of cheating) in column **(**1), the average number of puzzles reported as solved (average cheating) in column **(**2), and the average number of puzzles reported as solved only for cheaters (intensive margin of cheating) in column **(**3) for each treatment combination. Figures 4, 5 and 6 respectively present averages and 95 percent confidence intervals by treatment combination for dishonesty measures 1, 2 and 3. Using the average cheating measure, each of the means of treatment conditions is statistically different from both zero and 10 (maximal cheating) at the 1 percent level (Wilcoxon test, two-sided p-values are 0.0000 for all treatment combinations and both sets of tests). The highest extensive margin of cheating occurs for the P/S group (59 percent) while the highest intensive margin is found for the NP/NS group (2.74).

**Table 2:** Comparison of means across treatment combinations.

Treatment	Extensive Margin (Proportion cheating) (1)	Average Cheating (includes truthful report) (2)	Intensive Margin (Average Cheating for Cheaters) (3)
No Piece Rate/No Shred (NP/NS)	0.58	1.58	2.74
No Piece Rate/Shred (NP/S)	0.63	1.58	2.51
Piece Rate /No Shred (P/NS)	0.37	0.67	1.82
Piece Rate /Shred (P/S)	0.59	1.39	2.38

Contrasts	Dunn,Wald	Dunn,Wald	Dunn,Wald
(1) NP/NS vs. NP/S	–	–	–
(2) NP/NS vs. P/NS	**,**	***,***	**,**
(3) NP/NS vs. P/S	–	–	–
(4) NP/S vs. P/NS	***,***	***,***	**,*
(5) NP/S vs. P/S	–	–	–
(6) P/NS vs. P/S	***,***	***,***	*,–

‘*’, ‘**’, ‘***’ significant at the 10, 5 and 1 percent levels respectively, ‘– ‘ statistically insignificant.

Using only participants from our P sessions we find that average levels of cheating for P/S and P/NS are 1.39 and 0.67 respectively which are statistically different from zero and 10 as noted above. Furthermore, as for data aggregated over all sessions, for the P/S and P/NS sessions separately, the likelihood of a participant cheating by a small amount is significantly greater than the likelihood of cheating by medium or large amounts (Wilcoxon test, two-tailed p-values = 0.0000, 0.0000 respectively). Our results allow us to reject hypothesis 1 and we summarize the above findings more succinctly below:

*Result 1*: Participants who are paid piece-rate incentives on average cheat by a small amount that is significantly different from zero. However, they do not cheat maximally even when their dishonesty cannot be detected.

Using any of the measures: extensive margin, average cheating, or the intensive margin, the average level of dishonesty for our NP participants (measured separately for the S and NS groups or pooled) is significantly greater than zero at the 1 percent level (Wilcoxon test, two-sided p-value = 0.0000) allowing us to strongly reject hypothesis 2.

*Result 2:* The likelihood and magnitude of cheating is significantly greater than zero even in the absence of financial incentives.

Pooling over our S and NS groups, average cheating is significantly higher (Wilcoxon z-stat = 2.419, *p*-value = 0.0156) for the group that does not receive the payment of Rs.50 piece rate (NP). Qualitatively similar to our results, the effect of an increase in reward for cheating in the matrix task in Mazar et al. ^11^ and for the standard report of a die-roll in Kajackite and Gneezy^14^ show a decrease in the magnitude of reported outcomes (non-monotonically in the latter) though this decrease in both studies is not significant. Likewise, pooling over participants from the P and NP groups, when the probability of detection is decreased to zero (from NS to S), misreporting increases significantly (Wilcoxon z-stat = 2.283, *p*-value = 0.0224). Thus a-priori, dishonesty decreases in terms of average cheating as the amount one makes by cheating as well as the probability of getting caught increase. In the analysis to come we will see that controlling for levels of the other treatment variable and individual level attributes, decreasing the detection probability to zero is not significant in independently increasing the level of dishonesty for all groups in our sample.

Table 2 also compares the proportion who cheat and the average magnitude of cheating both overall and for only cheaters, among our four treatment conditions. We use ANOVA based Wald test contrasts as well as the non-parametric Dunn test^67^, which allows us to do intergroup comparisons, when the Kruskal–Wallis test for equality over multiple independent groups is rejected. Our procedure can be described as non-parametric ANOVA. In our case the non-parametric Kruskal–Wallis test returns a χ2 statistic (with ties) of 14.11 with 3 d.f. and a p-value = 0.0028 for averages, and 10.76 with 3 d.f. and a p-value = 0.0131 for proportions. Above we present only the significance of the different contrasts. Detailed test statistics for these may be found in Supplementary Tables S3–S5.

Notice from the contrasts above, that the effects of detection probability and piece-rate reward are not uniform even though overall both variables matter to determine cheating. Specifically, for the S (zero probability of detection) group, paying participants piece-rate or not does not change the proportion or level of cheating significantly (contrast 5). For the NS group (positive probability of detection), paying individuals piece-rate incentives lowers the proportion of individuals who cheat by about 20 percent and the level of cheating by more than half (contrast 2). Thus, increasing the piece-rate reward from zero to a positive value decreases dishonest behaviour significantly only when individuals’ dishonesty can be ex-post detected.

Turning our attention to the probability of detection, we find that for the group that does not receive piece-rate payments (NP), decreasing the probability of detection to zero does not significantly increase the proportion of cheaters and the cheating magnitude (contrast 1). On the other hand, for participants being paid piece-rate (P), decreasing the probability of detection to zero increases the proportion of individuals who cheat by approximately 20 percent and more than doubles the average level of cheating (contrast 6). For column [3], we find contrast 6 to be marginally significant only for the non-parametric contrast. This is probably due to the fact that there are only 22 individuals who report solving greater than 0 in the P/NS group.

Overall we see that allowing participants to shred their problem sheets increases dishonest behaviour significantly only when individuals are paid piece-rate rewards. The result that in the absence of piece rate incentives individuals really don’t care about the probability of detection, appears to be mirrored in Mazar et al.^11^ who find with a low piece-rate of $0.10, decreasing the probability of detection does not increase average cheating magnitude. This suggests that making the probability of detection zero matters only when there are substantial monetary consequences that arise from cheating. A real world parallel for cheating in the NP condition could be verifiable or unverifiable claims with negligible-financial stakes that individuals use in their resumes, such as playing up their association with famous individuals and institutions, over-hyping minor achievements or mildly overstating their qualifications. Accordingly, we conjecture that perhaps a participant reporting more in the NP treatment does not see this act as morally reprehensible even though in certain cases (the NS sessions) the experimenter can detect their dishonest act ex-post.

### Cheating in NP and NS treatments: affective benefits and social acceptability

We find significantly higher cheating in our combined NP treatments as compared to combined P treatments. In other words, participants in our experiment are overall more dishonest when the payment is independent of their self-report. In contrast, Charness et al.^17^ do not find significant difference in cheating in their treatment with flat payments. We conjecture that the divergence in results is due to the nature of the tasks chosen: ability-based task in our experiment as compared to the die-roll task in Charness et al.^17^. Though our study with a single type of task cannot be used to verify this, our results suggest that cheating to signal higher intellectual ability (even to oneself) may carry some intrinsic affective benefit. In contrast, when rewards are purely material (lying to misreport a random die roll) individuals appear to not be intrinsically motivated to lie when there is no actual financial reward that comes from it. Mazar et al.^11^ test whether or not an individual’s desire to not appear unintelligent compared to other participants drives their dishonesty behaviour, by manipulating their beliefs about the average task completion rate of others in their cohort. They find that exaggerating the latter does not significantly drive dishonesty. However our tasks are more intellectually sophisticated than the largely effort based matrix task used by Mazar et al.^11^ so it is possible that individuals may obtain an affective benefit or a “cheater’s high” from reporting that they solved more in the absence of piece-rate incentives as documented by Ruedy et al.^30^ using intelligence tests in the form of math or logic problems. Furthermore, it may be possible that individuals of lower academic achievement obtain greater affective benefit from lying about their intelligence in order to maintain their self-image. This effect may be giving rise to the small but statistically significant negative association between self-reported XIIth grade score and dishonesty (Table 4), though our study is not able to test this systematically.

**Table 3:** Variables used in regression analysis.

Variable	Description	Average	Min	Max
Dishonesty [D, Dependent]	Takes the value 1 if participant reported solving > 0, 0 otherwise	0.55	0	1
Magnitude of Dishonesty [MagD, Dependent]	The number of puzzles the participant reported to have solved	1.30	0	9
Age	Age in years	19.50	17	25
Female	Takes the value 1 if female, 0 otherwise	0.55	0	1
Piece-Rate [P]	Takes the value 1 if the participants are paid piece rate, 0 otherwise	0.5	0	1
Shred [S]	Takes the value 1 if the participants are allowed to shred their problem sheet, 0 otherwise	0.58	0	1
Marks12	Percentage marks secured in XII^th^ grade school board examination	86.84	57.40	97.25
Economic Satisfaction	Happy with the current economic situation prevailing in the family. Scale variable: 1. Not at all, 2. Not entirely 3. Yes	2.32	1	3
Peer Comparison	Own economic condition compared to peers. Scale variable: 1. Worse, 2. Same 3. Better	2.23	1	3

**Table 4:** Regression results.

Independent variables	Dep: MagD OLS (1)	Dep: D = 0/1 Binary Logit [Marginal Effects] (2)	Dep: MagD, given D = 1, Truncated OLS (3)
Piece Rate [P = 1]	– 0.97*** (0.28)	– 0.21** (0.09)	– 1.98*** (0.62)
Shred [S = 1]	0.09 (0.31)	0.04 (0.09)	0.32 (0.52)
Piece-Rate*Shred [P = 1 & S = 1]	0.92** (0.39)	0.19* (0.12)	1.69** (0.73)
Age	0.02 (0.09)	– 0.01 (0.03)	0.13 (0.13)
Female	0.20 (0.19)	0.10 (0.06)	– 0.21 (0.34)
Marks12	– 0.04*** (0.015)	– 0.006 (0.004)	– 0.10*** (0.03)
Eco. Satisfaction	– 0.37** (0.17)	– 0.04 (0.05)	– 0.82*** (0.30)
Peer Comparison	0.55*** (0.17)	0.13** (0.05)	0.47 (0.29)
Constant	4.27* (2.21)		9.07** (3.73)
Number of Observations	264	266	144
Pseudo *R*^2^/ *R*^2^	0.14	0.05	0.22

Robust standard errors in parentheses.

‘*’, ‘**’, ‘***’ significant at the 10, 5 and 1 percent levels respectively.

Like Friesen and Gangadharan^32^ we obtain that even when post-facto verification is possible, e. g.—our no-shred (NS) condition, a significant amount of over-reporting is observed. Though it is not possible for us to check the sheets the participants shredded, for NS participants, we check to see if they perhaps mistakenly assumed they had solved a puzzle or two and reported this without meaning to be dishonest. We find that all participants including those who report a positive amount had barely attempted the puzzles and had come nowhere close to finding any kind of solution. Thus, there clearly are individuals who are brazen enough to be willing to take small liberties with the truth even when it can be later verified. We would of course never know the identity of an individual as our procedure made clear to our participants. So, there was likely to be no significant reputational concerns that an individual could have had. However, a participant would be aware that we could recover the amount he or she cheated by comparing their self-report with their submitted puzzle sheet. The NP/NS group has average over-reporting of approximately 1.6 puzzles, matching that of the NP/S group. A particularly interesting group is that of individuals who cheat in the P/NS treatment. These 22 individuals actually lie to gain piece-rate incentives when their (mis)deed can be post-facto verified. Running an OLS regression only on the NS group (*n* = 116, *R*^2^ = 0.20, not reported here) we find that much like the overall regressions, piece-rate payments significantly lower the level of dishonesty, indicating that individuals are more careful when their action has actual material consequences to the experimenter.

### Peer comparison and economic satisfaction

The variables peer comparison and economic satisfaction try to capture extant subjective self-perceptions of one’s wealth state with respect to peers and wealth aspirations respectively. We purposely do not use self-reported absolute measures of income or wealth as close to 85 percent of our undergraduate student sample do not have any independent income and may not be able to accurately estimate parental or family earnings. We instead choose a measure of subjective economic status (SES) which tries to capture how wealthy an individual feels with respect to his or her peers. As a consistency check we explore how this subjective measure correlates with the responses to another question in our questionnaire that asks individuals to rank their family wealth on a seven-point scale going from No Wealth to Very Wealthy. We find that the peer comparison measure we use is positively and significantly correlated with this wealth ranking (p-value = 0.0000).

Our measure of aspiration that uses dissatisfaction with one’s current economic state is inspired by Ray^68^ and Genicot and Ray^69^, where aspirations are defined as the difference between the current economic standard and the future economic standard that a person wishes to achieve. According to Ray^68^, p.2, lines 20, 21) in defining aspirations, i.e.—where one wants to be economically in the future, “individuals use their peers (or near-peers) to form comparisons, invidious or otherwise.” Specifically, the variable that asks if an individual is happy with his or her current wealth situation tries to qualitatively interpret the “aspiration gap” which is zero if an individual feels completely satisfied (“yes”), is positive if he is “not entirely” satisfied and is high if he is “not at all” satisfied.

**Figure 7:**
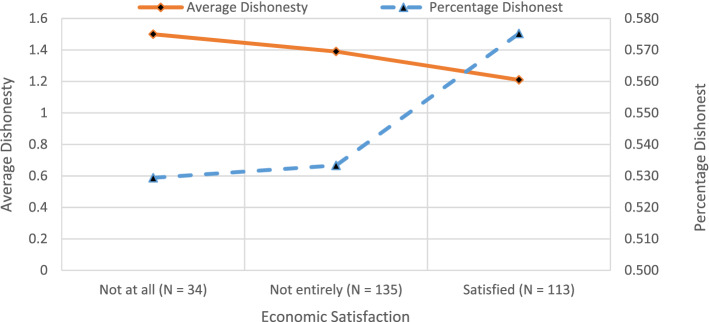
Likelihood and magnitude of dishonesty with economic satisfaction.

Supplementary Table S6 presents the likelihood (extensive margin) and magnitude of dishonesty (average cheating) with increasing economic satisfaction and SES for all our data as well as separately for treatments NP and P. Supplementary Table S7 presents three group Wald tests for differences in the likelihood and average magnitude of cheating for different levels of economic satisfaction and SES for the overall data as well as separately for treatments NP and P. Figure 7 shows the change in likelihood (extensive margin) and magnitude (average cheating) of dishonesty with increasing economic satisfaction. The proportion of individuals who cheat rises from about 53 percent for individuals who don’t feel satisfied to about 58 percent who do. The average level of dishonesty falls from about 1.5 to 1.21. However, we find from Supplementary Table S6 that the extensive margin (percentage) or average cheating for either the pooled data or the data split over our two reward schemes do not significantly differ with levels of economic satisfaction.

**Figure 8:**
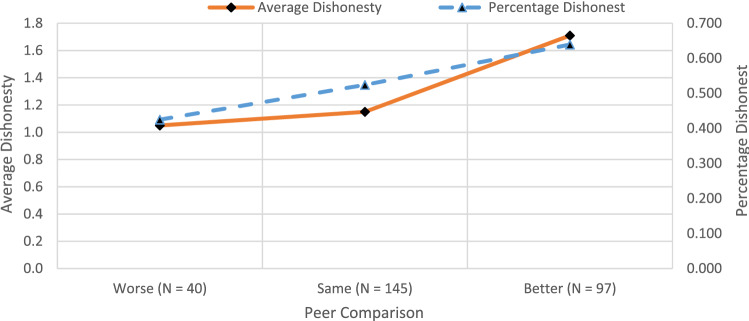
Likelihood and magnitude of dishonesty with relative wealth.

Figure 8 shows that both the proportion as well as the average magnitude of cheating are higher for individuals who feel that their economic condition is better than their peers. From the Wald contrasts reported for the pooled data in Supplementary Table S7 we find a significantly higher percentage of individuals who cheat among the group who feel richer than their peers as compared to those who feel that their economic condition is worse (Wald F-test *p*. value = 0.0220). Furthermore, the average magnitude of dishonesty for the group that feels richer than their peers is significantly higher than the group who report either feeling that their economic position is the same (Wald F-test *p*. value = 0.0103) or worse (Wald F-test *p*. value = 0.0339). The positive relationship between dishonesty and SES is also seen separately for treatments NP and P in Supplementary Table S6. In the next section, using regression analysis we establish that these two variables independently determine preferences for dishonesty (result 5) after we control for demographic attributes, reward levels and probability of detection.

### Determinants of cheating: regression approach

In this section we use regression techniques to control for individual specific attributes in order to check if our main effects on the likelihood and magnitude of cheating remain valid once these other attributes are factored in. The definitions for the dependent and explanatory variables are given in Table 3 and individual attributes are presented by treatment condition in Supplementary Table S2. Besides our two main treatment variables Shred [S = 1] and Piece Rate [P = 1] and their interaction [P = 1 and S = 1], the individual specific attributes we use are gender, age, self-reported academic ability, economic satisfaction and own economic condition compared to peers.

### One-stage regression models

A one-stage (one equation) specification is estimated as an OLS regression with robust standard errors. As there are a small number of outliers, who display levels of dishonesty that are over 4 times the average for all our participants (i.e.—dishonesty ≥ 5), the robust procedure^70–72^ adjusts the variance–covariance matrix for the effect of such outliers on linear estimation.

Table 4 reports coefficients from the OLS model in column (1). We find that when the probability of detection is positive [S = 0] introducing a piece-rate incentive of Rs. 50 [P = 1] for each question reported to be solved significantly lowers the level of dishonesty. On the other hand, the main effect of allowing participants to shred their sheets [S = 1] is insignificant. This indicates that when monetary incentives are absent [P = 0], allowing individuals to shred their task sheet has no effect on their cheating behaviour. However, the interaction between P and S [P = 1 and S = 1] is positive and significant. This indicates that compared to the condition where piece rate incentives are absent and detection probability is positive, introducing piece rate incentives and lowering the probability of detection to zero significantly increases the level of dishonesty. Furthermore, if we compare coefficients or marginal effects for our main effects [S = 1] and [P = 1] with the interaction, we see that in both cases: introducing piece rates for participants allowed to shred and decreasing detection probability to zero for those who earn piece-rate, dishonesty increases, verifying our results from the univariate analysis presented earlier.

Supporting the higher ability-lower dishonesty link obtained by Miller et al.^37^ and Ruffle and Tobol^38^, dishonesty in our experiment is seen to be significantly and negatively associated with self-reported XII^th^ grade score though the effect size is small. We obtain that individuals who feel economically superior in comparison to their peers cheat significantly more, supporting the findings of Piff et al.^41^. We also see that those who feel more satisfied with their current economic condition cheat significantly less. Age and gender are seen to have no effect on dishonesty.

### Two-stage or hurdle regression

We investigate the impact of our two main treatment variables (financial reward and probability of detection) along with other demographic, ability and wealth-related variables on the likelihood and magnitude of cheating using a hurdle regression approach (see^73^. In this approach, we model cheating as a two-stage data generation process. The first stage, which we estimate as binary logit, models the decision of whether or not to cheat and is what we describe earlier as the extensive margin for cheating. Conditional on deciding to cheat, the second stage models the magnitude of cheating using a truncated OLS regression, and this magnitude represents the intensive margin of cheating. As pointed out by Yaniv and Siniver^16^, the original SMORC^8,9^) does not model the number of dishonest acts by a perpetrator and simply identifies conditions under which an individual would commit a crime, i.e.- identifies the extensive margin of cheating. Our first stage studies this likelihood of committing a dishonest act without reference to the number of such acts committed. As before our regressions use robust standard errors.

Since logit regression coefficients represent the log odds ratio for the binary outcome, more meaningful estimates to present are marginal effects [d*p/*dX], i.e.—change in likelihood of cheating due to a unit change in an explanatory variable, which are shown in column (2) of Table 4. We find that increasing piece rate incentives from zero to Rs. 50 [P = 1], lowers the likelihood of cheating by about 22 percent for individuals who are not allowed to shred their sheets. However there is no effect of decreasing the probability of detection to zero [S = 1] on the likelihood of cheating if piece rate payments are absent. As in the single stage regressions, the interaction [P = 1 and S = 1] is positive and marginally significant. As an interaction in the logit model is difficult to interpret we also estimate this likelihood using a linear probability model (LPM). The estimates are given in column (1) of Supplementary Table S9 and are very similar to the logit marginal effects. Specifically, the size of the interaction is identical to that from the logit model, but like the latter it is not significant at the 5 percent level. Furthermore, a higher self-perception of wealth compared to one’s peers by one unit (from worse to same or from same to better) is associated with a higher likelihood of cheating by 13 percent controlling for other variables at the 5 percent level of significance.

From the truncated OLS estimates in column (3), we find as before that the incidence of cheating significantly decreases in the piece-rate condition [P = 1] for individuals with a positive probability of detection. Furthermore, dishonesty significantly increases for the group that is paid piece-rate and allowed to shred their sheets [P = 1 and S = 1] as compared to individuals who are not paid piece-rate and have a positive probability of detection. Similar to the first stage, there is no significant effect of allowing participants to shred their sheets [S = 1] when piece-rate payments are absent [P = 0]. As in the one stage regression, self-reported XII^th^ grade score negatively affects the magnitude of dishonesty. Finally, individuals who report feeling satisfied with their current economic condition by one unit more (from “not at all” to “not entirely”, or from “not entirely” to “satisfied”) report having solved approximately one question less than others. Thus a lowering of the aspiration gap (as interpreted from^68^ is associated with a reduced magnitude of cheating.

Our single hurdle model makes a strong assumption, i.e.—the errors of the first and second stage regressions are independent. A more plausible scenario may be that the errors are correlated, necessitating a double-hurdle model as first introduced by Cragg^74^. We use a formulation of the double hurdle model as posited by Engel and Moffatt^75^ that specifies the first step as probit and the second as Tobit. These estimates are given in columns (2) and (3) of Supplementary Table S9. Comparing results from our single hurdle model given in columns (2) and (3) of Table 4 with the appropriate columns from Supplementary Table S9 we find that the effects of our main variables of interest (incentives, detection probability) remain close in size and identical in sign and significance. The only difference in significance that we observe is for the variable Economic Satisfaction where the level of significance in the second stage drops from 5 to 10 percent.

### Robustness: institution level heterogeneity

To check the robustness of our main effects we also take into account inter-institution variability in dishonesty. We have the option of fitting fixed or random effects models that allow us to account for this in our main effects. The Hausman Specification Test returns a χ^2^ statistic of 19.72 with a p. value of 0.0062, indicating that a fixed effects formulation may be more appropriate. However given that one of the treatment arms of our experiment, i.e.—probability of detection, has only the shred [S = 1] condition in some institutions and only the no-shred [S = 0] in all others, running a fixed effects specification would lead to the shred variable being dropped due to collinearity and non-identification of one of the arms of our experiment. In lieu of this we run a least squares dummy variables (LSDV) specification incorporating 9 institutional dummy variables. The hurdle model used is of the single hurdle type and employs a Tobit specification for the second stage estimation. The regression procedure in STATA omits two institutional dummies in each regression in order to allow for both arms of the experiment to be identified. These estimates are given in Supplementary Table S8.

Table 5 reports results from specifications that use institutional random effects, a single hurdle and a Tobit specification for the second stage. Comparing the appropriate regressions from Table 4 with those from Table 5 (and Supplementary Table S8) we find that variables pertaining to our main variables of interest continue to be similar in sign and significance, possibly informing us that our participant pool even though from several colleges and universities is quite homogenous.

**Table 5:** Regression results with College/Univ Random Effects.

Independent variables	Dep: MagD OLS (1)	Dep: D = 0/1 Binary Random Effects Logit [Coefficients] (2)	Dep: MagD, given D = 1, Random Effects Tobit (3)
Piece Rate [P = 1]	– 0.97*** (0.12)	– 0.92** (0.40)	– 1.74*** (0.53)
Shred [S = 1]	0.09 (0.26)	0.07 (0.48)	0.17 (0.55)
Piece-Rate*Shred [P = 1, S = 1]	0.92*** (0.25)	0.83 (0.53)	1.61** (0.68)
Age	0.02 (0.097)	– 0.0003 (0.12)	0.04 (0.14)
Female	0.20 (0.21)	0.46 (0.28)	0.49 (0.36)
Marks12	– 0.04*** (0.01)	0.0005 (0.026)	– 0.05 (0.03)
Eco. Satisfaction	– 0.37*** (0.13)	– 0.15 (0.21)	– 0.51* (0.27)
Peer Comparison	0.55*** (0.20)	0.52** (0.21)	0.88*** (0.27)
Constant	4.27 (2.72)	– 0.67 (3.80)	3.16 (4.33)
Number of Observations	264	266	144
Pseudo *R*^2^/ *R*^2^	0.14		–

Standard errors in parentheses (Robust s. e. adjusted in 9 clusters for OLS).

‘*’, ‘**’, ‘***’ significant at the 10, 5 and 1 percent levels respectively.

Panel Tobit models do not report a goodness of fit measure.

The self-reported school leaving score: Marks12 and Economic Satisfaction continue to be significant in the single stage models. However the former drops out of significance while the latter is significant only at the 10 percent level in the hurdle regression models. Furthermore, there are no effects significant at the 5 percent level in Tables 5 and Supplementary Table S8 which are insignificant or marginally significant in Table 4.

We summarize below the important findings related to incentives and probability of detection from our regression analysis in results 3 and 4. Results 3 and 4 thus allow us to reject both hypotheses 3 and 4.

*Result 3*: Introducing piece-rate incentives significantly decreases both the likelihood and magnitude of cheating only for individuals who face a positive probability of detection.

*Result 4*: Decreasing the probability of detection of cheating to zero significantly increases the magnitude of cheating only for the group paid piece-rate incentives.

Results 3 and 4 taken together also show us that the effect on dishonesty of either material incentives or probability of detection is not independent of the other but that these motivations interact. Our results indicate that some form of moral or ethical curb on cheating is present when piece rate incentives are introduced for only some of our participants who have a positive probability of detection. However, it is important to mention that standard economic rationality operates too and increases dishonesty when detection is impossible, albeit for only one set of participants: those that are paid piece-rate incentives.

The regression approach strengthens our results from the univariate tests in the earlier sub-section on peer comparison and economic satisfaction, which are both seen to independently determine preferences for cheating. This allows us to reject hypotheses 5 and 6. We summarize this analysis below in result 5:

*Result 5*: Feeling wealthier than one’s peers is associated with a significantly higher likelihood of cheating, whereas feeling more satisfied with one’s current economic state is associated with a significantly lower magnitude of cheating.

As we have undergraduate students from many disciplines some of which are under-represented in our sample, it is not possible for us to explore whether there are course specific norms with respect to cheating. For groups which have larger representation in our sample: participants who study economics (n = 72) and the combined group of participants who study economics or business (n = 146) do not display higher average magnitude of cheating than participants from other disciplines (t-test *p*-values = 0.3607 and 0.1718 respectively).

## Discussion and conclusion

We conduct an experiment to explore the effects of financial incentives and probability of detection on the level of dishonesty measured at the individual level. Our measure of dishonesty is obtained from the over reporting of the number of real-effort puzzles solved out of ten. Since all of the puzzles are unsolvable, any positive number of puzzles reported as solved is considered as dishonest reporting. The unsolvable nature of the puzzles is unknown to our participants.

Maximal cheating as reported by Yaniv and Siniver^16^ is not seen in our experiment. When the threat of detection is zero (or very marginal even to an abnormally fearful individual) and there are substantial piece rate incentives to cheat, the average level of cheating among our participants is only about 14 percent. In fact regardless of the treatment condition the average level of over-reporting never exceeds 16 percent, keeping the level of dishonesty in the range obtained by Mazar et al.^11^. In our experiment, increasing financial incentives appears to act as a moral curb and lowers the level of observed dishonesty. Our results thus lend more support to moral balance and self-concept maintenance theories in Nisan^18^ and Mazar et al.^11^ respectively, rather than rational theories of dishonesty such as^8,9^. However extensions of Becker’s simple model of rational crime by incorporating individual attributes such as risk aversion and moral curbs like shame as done by Yaniv and Siniver^16^ may potentially also explain the behaviour of our participants.

We deconstruct the cheating decision into two processes, i.e.—the binary decision to cheat or not and the magnitude of cheating conditional on deciding to cheat. We find that introducing piece-rate incentives significantly lowers both the likelihood and magnitude of cheating only in the treatment condition where there is a positive probability of detection. Individuals who are allowed to shred their sheets do not display differential incentive effects. On the other hand, decreasing the probability of detection of cheating to zero significantly increases the magnitude of cheating only for the group that is paid piece-rate financial incentives and not others. Additionally, participants in our experiment are significantly dishonest even when there are no piece-rate incentives offered to them. This suggests that there may be non-monetary or affective gains in utility associated with cheating over and above incremental monetary benefits.

Among individual specific variables, self-reported XII^th^ grade academic performance is associated with an increased magnitude of cheating. Additionally, feeling wealthy compared to peers is associated with increased likelihood of cheating, while increased economic satisfaction is associated with a lower magnitude. The fact that these last two variables are independently significant implies that at any level of own wealth perception vis-à-vis one’s peers, economic dissatisfaction is associated with higher cheating. Thus we may cautiously make the case that wealth aspirations of unsatisfied individuals could be an additional independent factor driving dishonesty, making richer individuals more prone to cheating as seen in Piff et al.^41^ in contexts different to ours. In this study we do not really pursue in-depth the interaction between SES and economic satisfaction in driving dishonesty but it may be an interesting area for follow-up research especially in the context of a developing country like India.

Finally, our results point to at least two interesting directions for future research. First, we observe that individuals lie significantly in problem solving experiments like ours and those of Ruedy et al.^30^ when they are not paid piece-rate rewards but not in the die-roll experiments of Charness et al.^17^. This indicates that the task context may be important in determining non-monetary benefits of cheating independent of financial rewards. In addition, the fact that quite a few individuals in our sample lie about their performance to gain financial rewards even when this can be post-facto verified may suggest that social norms regarding what constitutes an acceptable task-specific level of dishonesty are at play. Banerjee^76^ finds that the moral framing of objectively equivalent tasks determines acceptable levels of dishonesty in the laboratory. Future research could explore social acceptability of cheating in a systematic manner with different cheating contexts and their associated moral frames. Such experiments would also offer a direction in which existing theories like those of self-concept maintenance may be extended. These theories posit that there is an individual specific acceptability threshold up to which a person cheats (in terms of both monetary and affective utility) beyond which he lowers his self-image. Results from Charness et al.^17^ and our study suggest that that this threshold may be sensitive to task contexts that make us both perceive and aggregate affective and financial benefits accruing from dishonest acts in a differential manner. This may also suggest that there are no intrinsically “honest” individuals, and a person may choose to be significantly dishonest in one context and perfectly honest and ethical in others.

Second, as Kajackite and Gneezy^14^ also conjecture, it may be possible that paying a substantial piece-rate payment (more serious consequences) makes cheating look criminal and hence is more deterring in the minds of the subjects. This implies theoretically, that an increase in the rewards from cheating may trigger two opposing effects, one of which is the economic motivation to increase cheating while the other is in the form of moral accountability that limits cheating. These motivations may be independent of actual detection probability. In the context of India, a developing country with both high inequality^77^ and a high corruption perception index^78^, we conjecture that perhaps the affective deterrence associated with obtaining someone else’s resources by unfair means is substantial enough to overcome economic benefits and strongly limit dishonesty as seen in our experimental participants. Furthermore, we see in our data that subjective perceptions of own wealth, which are determined and modified by context, correlate with dishonesty. Future research could examine the link between socioeconomic contexts and the perceived moral accountability of the consequences arising from dishonesty in a systematic manner.

## Supplementary Information

Supplementary Information.
