# Supplementary material for: Experimental evidence of the effect of financial incentives and detection on dishonesty
Source: Sci Rep. 2022 Feb 17;12:2680. doi: 10.1038/s41598-022-06072-3 (PMC8854596; doi:10.1038/s41598-022-06072-3)
Supplement: Supplementary file 1 — Supplementary Information. [file 41598_2022_6072_MOESM1_ESM.docx]

**Supplementary Information**

Title: Experimental Evidence of the Effect of Financial Incentives and Detection on Dishonesty.

Authors: Mehak Kaushik, Varsha Singh, Sujoy Chakravarty

**Instructions and Puzzle Tasks**

**THE BRAIN TEASERS**

**Here is a chance to exercise your brain over some puzzles and earn some more money!**

Designed to determine spatial-logic abilities, problem solving skill, concentration power and innovative thinking

These questions test fundamental requirements for management, research abilities and almost everything. ☺

You will be presented with **10 puzzles**.

You will be given **5 minutes** to complete the entire test. All questions are compulsory. There are no options.

**In this task, you will receive Rs.50 for each puzzle that you solve. Your earnings during this task will be added to your participation reward of Rs.250. For example, if you solved ‘X’ number of puzzles, you will receive Rs.250 + 50*X. [For NP sessions this paragraph is not present]**

Please note that **no questions will be entertained during the test**. If you have any questions, you may raise your hand now.

Else, please wait for further instructions about the test.

***THE PUZZLE SHEET***

**Q1:** Rearrange **exactly two** balls in figure (i) so that the triangular pattern points down (figure (ii)) instead of up.


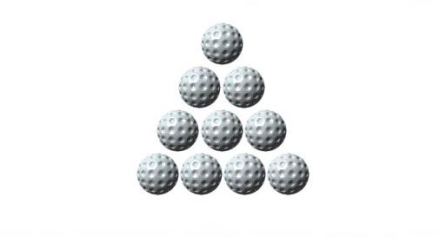

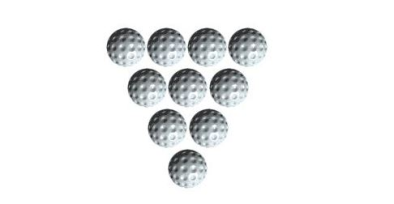


(i) (ii)

**Space for working:**

**Q2:** You have to form **4 equilateral triangles** (triangles with all sides equal) using **only 6 sticks of same length** (as shown below). All triangles should be of same size.

**Note: Overlapping or breaking the sticks is not allowed.**


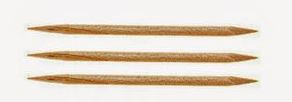

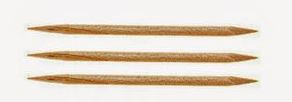


**Draw your answer in the space provided below.**

**Space for working:**

**Q3:** You are given a rectangular figure. This figure is divided into 5 rooms. Trace a **continuous path** (without lifting the pen) passing through **each door** (drawn in bold lines) **only once.**


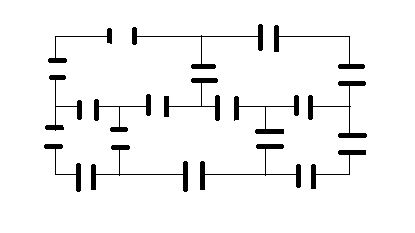


**Space for working:**

**Q4**: Connect all nine dots using **only three straight lines**, without removing your pen from the paper.


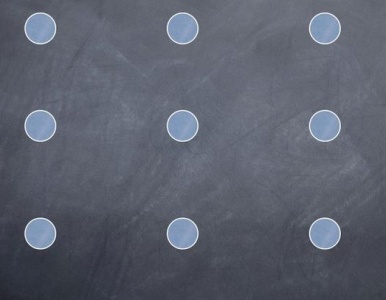


**Space for working:**

Questions 5 to 8 are mazes in which you trace a way from a point of entry to an exit. The entry point is marked by
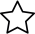
 and the exit point is marked by #. Trace a path for each of them.


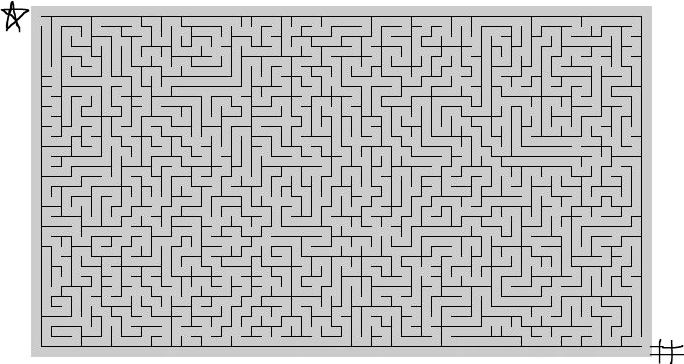
**Q5:**


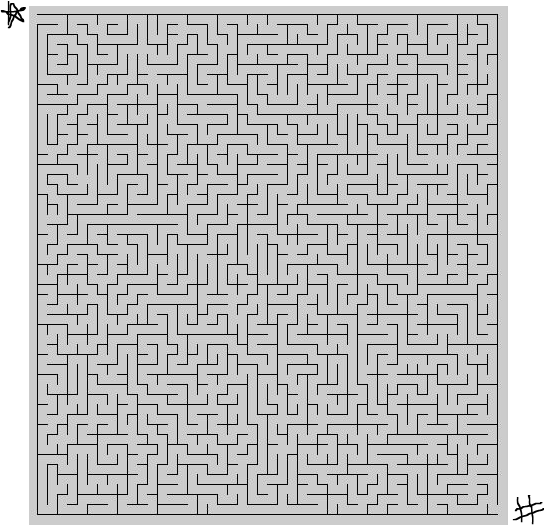
**Q6:**


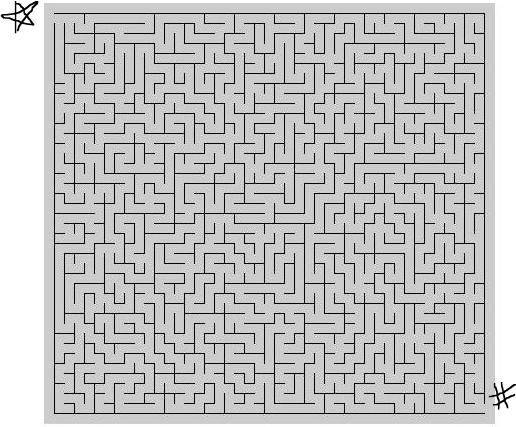
**Q7:**


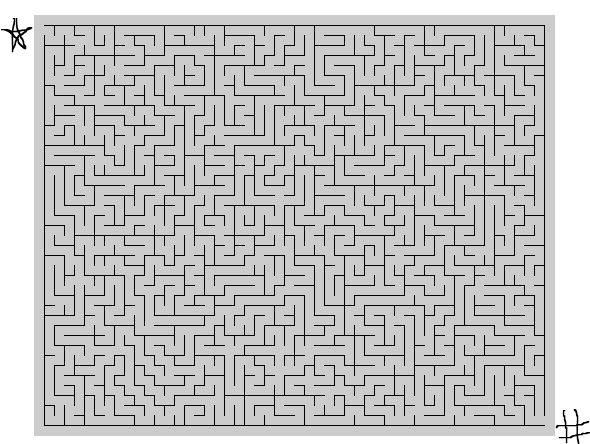
**Q8:**

**Q9:** There are five squares (one 3x3 and four 1x1) formed with 20 matchsticks as shown in the illustration. Move **exactly two matchsticks** to form **eight squares.**

**Note:** Overlapping, breaking the match sticks or ‘loose ends’ are not allowed.


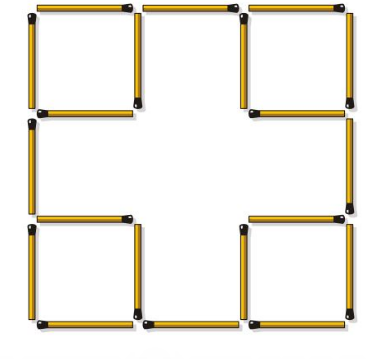


**Space for working:**

**Q10:** Alice has bought a large doughnut (it has a hole in the middle and only the coloured portion is eatable, as shown in the figure below), to share with her friends. She invited 11 people to her house. Fortunately, none of Alice’s friends mind how much they get as long as they get something from the doughnut. Using **exactly 3 straight lines,** how can Alice divide the doughnut into **12 pieces**?

**Space for working:**

SELF- REPORT CARD

**ID NO.________________**

Please write your ID number in the space provided.

You are **not required** to submit your ‘puzzle sheet.’ You are only required to report the number of puzzles you could solve in the space provided below.

[For NS sessions the above sentence is replaced with “You are required to report the number of puzzles you could solve in the space provided below.”]

**No. of puzzles I could solve out of 10:**

**[For NS sessions, the phrase “Please hand over the puzzle sheet to the experimenter,” is inserted here.]**

**Please hand over this sheet to the experimenter and collect your payment.**

| **Task Question** | **Source** |
| --- | --- |
| 1 | Fropky.com: https://www.fropky.com/rearrange-golf-balls-for-triangular-pattern-vt54768.html  Modified by authors to be unsolvable using MS Word/Paint |
| **2** | https://www.stem.org.uk/resources/elibrary/resource/422047/six-toothpicks-problem  Modified by authors to be unsolvable using MS Word/Paint |
| **3** | https://en.wikipedia.org/wiki/Five_room_puzzle  Modified by authors to be unsolvable using MS Word/Paint |
| **4** | Mikevanhoozer.com https://mikevanhoozer.com/break-out-of-the-box/  Modified by authors to be unsolvable using MS Word/Paint |
| **5** | https://mazegenerator.net  Modified by authors to be unsolvable using MS Word/Paint |
| **6** | https://mazegenerator.net  Modified by authors to be unsolvable using MS Word/Paint |
| **7** | https://mazegenerator.net  Modified by authors to be unsolvable using MS Word/Paint |
| **8** | https://mazegenerator.net  Modified by authors to be unsolvable using MS Word/Paint |
| **9** | https://www.mathsisfun.com/puzzles/matchstick-frame.html  Modified by authors to be unsolvable using MS Word/Paint |
| **10** | https://www.mathsisfun.com/puzzles/horace-and-the-doughnut.html  Modified by authors to be unsolvable using MS Word/Paint |
|  |  |
| **PPP US$ to INR Ex. rate** | https://data.worldbank.org/indicator/pa.nus.ppp |

Table S1: Source of puzzle task images and the US$ to INR exchange rate

| **Variable** | **Treatment NP/NS** | **Treatment**  **NP/S** | **Treatment**  **P/NS** | **Treatment**  **P/S** |
| --- | --- | --- | --- | --- |
|  |  |  |  |  |
| Age | 19.26 | 19.29 | 19.42 | 19.28 |
|  |  |  |  |  |
| Female | .38 | .54 | .44 | .56 |
|  |  |  |  |  |
| Marks12 | 83.55 | 87.62 | 81.66 | 86.72 |
|  |  |  |  |  |
| Economic Satisfaction | 2.29 | 2.33 | 2.17 | 2.31 |
|  |  |  |  |  |
| Peer Comparison | 2.15 | 2.28 | 2.12 | 2.22 |
|  |  |  |  |  |
| Number of participants | 60 | 82 | 60 | 82 |

Table S2: Average values of demographic variables broken up over treatment groups

| **Contrast (4 group)** | **Proportion who cheated** | **Wald test (F) stat** | **Wald p-value** | **Dunn test (χ2) stat** | **Dunn p-value** |
| --- | --- | --- | --- | --- | --- |
|  |  |  |  |  |  |
| (1) NP/NS vs. NP/S | NP/NS: 0.58 \| NP/S: 0.63 | 0.40 | 0.5263 | -0.63 | 0.2658 |
|  |  |  |  |  |  |
| (2) NP/NS vs. P/NS | NP/NS: 0.58 \| P/NS: 0.37 | 5.41 | 0.0207** | 2.29 | 0.0109** |
|  |  |  |  |  |  |
| (3) NP/NS vs. P/S | NP/NS: 0.58 \| P/S: 0.59 | 0.01 | 0.9137 | -0.11 | 0.4574 |
|  |  |  |  |  |  |
| (4) NP/S vs. P/NS | NP/S: 0.63 \| P/NS: 0.37 | 9.87 | 0.0019*** | 3.10 | 0.0010*** |
|  |  |  |  |  |  |
| (5) NP/S vs. P/S | NP/S: 0.63 \| P/S: 0.59 | 0.33 | 0.5658 | 0.57 | 0.2854 |
|  |  |  |  |  |  |
| (6) P/NS vs. P/S | P/NS: 0.37 \| P/S: 0.59 | 6.86 | 0.0093*** | -2.58 | 0.0049*** |
|  |  |  |  |  |  |
| ‘*’, ‘**’, ‘***’ significant at the 10, 5 and 1 percent levels respectively | | | | | |

Table S3: Treatment comparisons for the proportion who cheated (extensive margin)

| **Contrast (4 group)** | **Average Cheating** | **Wald test (F) stat** | **Wald p-value** | **Dunn test (χ2) stat** | **Dunn p-value** |
| --- | --- | --- | --- | --- | --- |
|  |  |  |  |  |  |
| (1’) NP/NS vs. NP/S | NP/NS: 1.576 \| NP/S: 1.580 | 0 | 0.9887 | -0.36 | 0.3579 |
|  |  |  |  |  |  |
| (2’) NP/NS vs. P/NS | NP/NS: 1.576 \| P/NS: 0.67 | 9.16 | 0.0027*** | 2.9 | 0.0019*** |
|  |  |  |  |  |  |
| (3’) NP/NS vs. P/S | NP/NS: 1.576 \| P/S: 1.39 | 0.44 | 0.5068 | 0.27 | 0.3908 |
|  |  |  |  |  |  |
| (4’) NP/S vs. P/NS | NP/S: 1.58 \| P/NS: 0.67 | 10.71 | 0.0012*** | 3.49 | 0.0002*** |
|  |  |  |  |  |  |
| (5’) NP/S vs. P/S | NP/S: 1.58 \| P/S: 1.39 | 0.55 | 0.46 | 0.7 | 0.242 |
|  |  |  |  |  |  |
| (6’) P/NS vs. P/S | P/NS: 0.67 \| P/S: 1.39 | 6.75 | 0.0099*** | -2.85 | 0.0022*** |
|  |  |  |  |  |  |
| ‘*’, ‘**’, ‘***’ significant at the 10, 5 and 1 percent levels respectively | | | | | |

Table S4: Treatment comparisons for average magnitude of cheating (both cheaters and non-cheaters)

| **Contrast (4 group)** | **Average Cheating** | **Wald test (F) stat** | **Wald p-value** | **Dunn test (χ2) stat** | **Dunn p-value** |
| --- | --- | --- | --- | --- | --- |
|  |  |  |  |  |  |
| (1’) NP/NS vs. NP/S | NP/NS: 2.74 \| NP/S: 2.51 | 0.43 | 0.5116 | 0.52 | 0.3009 |
|  |  |  |  |  |  |
| (2’) NP/NS vs. P/NS | NP/NS: 2.74 \| P/NS: 1.82 | 4.69 | 0.0319** | 2.26 | 0.0118** |
|  |  |  |  |  |  |
| (3’) NP/NS vs. P/S | NP/NS 2.74: \| P/S: 2.38 | 1.08 | 0.3008 | 0.88 | 0.1885 |
|  |  |  |  |  |  |
| (4’) NP/S vs. P/NS | NP/S: 2.51 \| P/NS: 1.82 | 3.07 | 0.0819* | 1.97 | 0.0242** |
|  |  |  |  |  |  |
| (5’) NP/S vs. P/S | NP/S: 2.51 \| P/S: 2.38 | 0.19 | 0.6656 | 0.41 | 9.3407 |
|  |  |  |  |  |  |
| (6’) P/NS vs. P/S | P/NS: 1.82 \| P/S: 2.38 | 1.95 | 0.1645 | -1.64 | 0.0510* |
|  |  |  |  |  |  |
| ‘*’, ‘**’, ‘***’ significant at the 10, 5 and 1 percent levels respectively | | | | | |

Table S5: Treatment comparisons for average magnitude of cheating (intensive margin: only cheaters)

|  | | **Pooled** | | **NP** | | | **P** | |
| --- | --- | --- | --- | --- | --- | --- | --- | --- |
| **Eco Satisfaction** | | **% Dishonest** | **Av. Dishonesty** | | **% Dishonest** | **Av. Dishonesty** | **% Dishonest** | **Av. Dishonesty** |
| Not At All | | 0.529 | 1.5 | | 0.67 | 1.93 | 0.42 | 1.16 |
| Not Entirely | | 0.533 | 1.39 | | 0.56 | 1.61 | 0.51 | 1.17 |
| Satisfied | | 0.575 | 1.21 | | 0.64 | 1.46 | 0.5 | 0.94 |
| **Peer comparison** |  | |  | |  |  |  |  |
| Worse | 0.43 | | 1.05 | | 0.57 | 1.35 | 0.24 | 0.65 |
| Same | 0.52 | | 1.15 | | 0.52 | 1.19 | 0.53 | 1.12 |
| Better | 0.64 | | 1.71 | | 0.73 | 2.11 | 0.52 | 1.19 |

Table S6: Percentage Dishonest and Average Dishonesty by levels of SES and economic satisfaction

| **Wald three-group Contrasts** | **Overall (N = 282)** | **Trt. NP (N = 140)** | **Trt. P (N=142)** |
| --- | --- | --- | --- |
|  | **Wald F, p-value** | **Wald F, p-value** | **Wald F, p-value** |
| **Percentage Dishonest** |  |  |  |
| Eco Sat. =1 vs. Eco. Sat. = 2 | 0.00, 0.9674 | 0.57, 0.4250 | 0.43, 0.5107 |
| Eco Sat. =2 vs. Eco. Sat. = 3 | 0.43, 0.5115 | 0.90, 0.3451 | 0.01, 0.9371 |
| Eco Sat. =1 vs. Eco. Sat. = 3 | 0.22, 0.6397 | 0.03, 0.8739 | 0.34, 0.5584 |
|  |  |  |  |
| **Average Dishonesty** |  |  |  |
| Eco Sat. =1 vs. Eco. Sat. = 2 | 0.13, 0.7206 | 0.40, 0.5267 | 0.00, 0.9673 |
| Eco Sat. =2 vs. Eco. Sat. = 3 | 0.66, 0.4181 | 0.21, 0.6466 | 0.70, 0.4031 |
| Eco Sat. =1 vs. Eco. Sat. = 3 | 0.77, 0.3798 | 0.83, 0.3631 | 0.28, 0.5960 |
|  |  |  |  |
| **Percentage Dishonest** |  |  |  |
| Peer Comp. =1 vs. Peer Comp. = 2 | 1.26, 0.2629 | 0.17, 0.6783 | 4.98, 0.0272** |
| Peer Comp. =2 vs. Peer Comp. = 3 | 3.14, 0.0774* | 5.55, 0.0198** | 0.00, 0.9465 |
| Peer Comp. =1 vs. Peer Comp. = 3 | 5.31, 0.0220** | 1.82, 0.1794 | 4.09, 0.0450** |
|  |  |  |  |
| **Average Dishonesty** |  |  |  |
| Peer Comp. =1 vs. Peer Comp. = 2 | 0.12, 0.7304 | 0.13, 0.7194 | 1.40, 0.2381 |
| Peer Comp. =2 vs. Peer Comp. = 3 | 6.68, 0.0103** | 7.93, 0.0056*** | 0.06, 0.8058 |
| Peer Comp. =1 vs. Peer Comp. = 3 | 4.54, 0.0339** | 3.05, 0.0830* | 1.59, 0.2100 |
|  |  |  |  |
| ‘*’, ‘**’, ‘***’ significant at the 10, 5 and 1 percent levels respectively  Notes: Eco Sat.: 1 = Not at all, 2=Not entirely, 3 =Satisfied. Peer Comp.: 1 = Worse, 2 = Same, 3 = Better | | | |

Table S7: Three group Wald tests exploring the association between dishonesty and aspirational variables

| **Independent variables** | **Dep: MagD**  **OLS**  **(1)** | **Dep: D= 0/1**  **Binary Logit**  **[Coefficients]**  **(2)** | **Dep: MagD, given D=1, Tobit**  **(3)** |
| --- | --- | --- | --- |
|  |  |  |  |
| Piece Rate [P =1] | -0.95***  (0.27) | -0.95**  (0.42) | -1.73***  (0.53) |
| Shred [S=1] | -1.02*  (0.52) | -0.86  (0.65) | -0.56  (0.78) |
| Piece-Rate*Shred  [P=1, S=1] | 0.90**  (0.39) | 0.88  (0.54) | 1.61**  (0.67) |
|  |  |  |  |
| Age | 0.07  (0.11) | 0.10  (0.14) | 0.14  (0.17) |
| Female | 0.32  (0.21) | 0.54*  (0.31) | 0.60  (0.36) |
| Marks12 | -0.02  (0.02) | 0.04*  (0.02) | -0.01  (0.03) |
| Eco. Satisfaction | -0.38**  (0.16) | -0.15  (0.22) | -0.49*  (0.27) |
| Peer Comparison | 0.54***  (0.17) | 0.54**  (0.22) | 0.86***  (0.27) |
| Constant | 1.94  (2.63) | -5.51  (3.52) | -2.47  (4.33) |
|  |  |  |  |
| Number of Observations | 264 | 266 | 144 |
| Pseudo *R*^2^/ *R*^2^ | 0.18 | 0.11 | 0.06 |
| Notes: Robust standard errors in parentheses  ‘*’, ‘**’, ‘***’ significant at the 10, 5 and 1 percent levels respectively  Two institutional dummies in each regression are omitted due to collinearity | | | |

Table S8: Regression results with College/Univ LSDV Dummies (not displayed)

| **Independent variables** | **Dep: D = 0/1**  **Linear Prob. Model (OLS)**  **(1)** | **Dep: D= 0/1**  **Probit**  **[Coefficients]**  **(2)** | **Dep: MagD, given D=1**  **Tobit**  **(3)** |
| --- | --- | --- | --- |
|  |  |  |  |
| Piece Rate [P =1] | -0.21**  (0.09) | -0.55**  (0.24) | -1.75***  (0.53) |
| Shred [S=1] | 0.04  (0.09) | 0.10  (0.23) | 0.23  (0.48) |
| Piece-Rate*Shred  [P=1, S=1] | 0.19  (0.12) | 0.51  (0.32) | 1.62**  (0.69) |
|  |  |  |  |
| Age | -0.01  (0.02) | -0.02  (0.06) | 0.01  (0.13) |
| Female | 0.10  (0.06) | 0.27*  (0.16) | 0.43  (0.34) |
| Marks12 | -0.01  (0.004) | -0.01  (0.01) | -0.06***  (0.02) |
| Eco. Satisfaction | -0.03  (0.05) | -0.08  (0.13) | -0.50*  (0.27) |
| Peer Comparison | 0.12**  (0.05) | 0.32**  (0.13) | 0.89***  (0.27) |
| Constant | 0.97  (0.62) | 1.12  (1.60) | 4.99  (3.40) |
|  |  |  |  |
| Number of Observations | 266 | 264 | 264 |
| Pseudo *R*^2^/ *R*^2^ | 0.07 | 0.05 | 0.04 |
| Notes: Standard errors in parentheses (Robust s. e. for LPM) | | | |
| ‘*’, ‘**’, ‘***’ significant at the 10, 5 and 1 percent levels respectively | | | |

Table S9: Robustness Regressions (LPM and Double Hurdle Model)
